# Comparison of Initialization Techniques for the Accurate Extraction of Muscle Synergies from Myoelectric Signals via Nonnegative Matrix Factorization

**DOI:** 10.1155/2018/3629347

**Published:** 2018-05-08

**Authors:** Mumtaz Hussain Soomro, Silvia Conforto, Gaetano Giunta, Simone Ranaldi, Cristiano De Marchis

**Affiliations:** Department of Engineering, Roma Tre University, Via Vito Volterra 62, 00146 Rome, Italy

## Abstract

The main goal of this work was to assess the performance of different initializations of matrix factorization algorithms for an accurate identification of muscle synergies. Currently, nonnegative matrix factorization (NNMF) is the most commonly used method to identify muscle synergies. However, it has been shown that NNMF performance might be affected by different kinds of initialization. The present study aims at optimizing the traditional NNMF initialization for data with partial or complete temporal dependencies. For this purpose, three different initializations are used: random, SVD-based, and sparse. NNMF was used to identify muscle synergies from simulated data as well as from experimental surface EMG signals. Simulated data were generated from synthetic independent and dependent synergy vectors (i.e., shared muscle components), whose activation coefficients were corrupted by simulating controlled degrees of correlation. Similarly, EMG data were artificially modified, making the extracted activation coefficients temporally dependent. By measuring the quality of identification of the original synergies underlying the data, it was possible to compare the performance of different initialization techniques. Simulation results demonstrate that sparse initialization performs significantly better than all other kinds of initialization in reconstructing muscle synergies, regardless of the correlation level in the data.

## 1. Introduction

The human neuromuscular system is known to be characterized by a highly complex structure, and the mechanisms adopted by the CNS for the generation of movement are not completely known [1]. The most common model for redundancy control hypothesizes that the CNS modulates muscle activity through a modular architecture [2]. In this model, it is possible to define a set of activation patterns of muscular groups (muscle synergies) that are used by the CNS to generate the coordination needed for a particular biomechanical task [3]. According to the theory of motor control through synchronous muscle synergies, muscle coordination can be represented by a pair of components: a spatial (**W**) and a temporal (**C**) one. **W** indicates the fixed balance of activation of a group of muscles and **C** the time-varying activation of each group.

Modular motor control strategies have been investigated in a variety of motor acts [4–11], and evidence has been provided that a set of muscle synergies is able to represent the control strategies underlying the movement, with motor modules that can be either typical for a particular task or shared among different tasks. These studies also revealed how the analysis of motor modules in conjunction with mechanical measurements can provide a neuromechanical description of human movement [12] and that it can be proposed as a means for quantifying motor impairment and planning neurorehabilitation [13, 14].

Various methods of estimating the spatiotemporal structure of synergies according to the synchronous model are discussed in [15]. Muscle synergies are extracted from multimuscle surface electromyographic (sEMG) recordings, by using various dimensionality reduction methods such as factor analysis (FA) [16], principal component analysis (PCA), independent component analysis (ICA), and nonnegative matrix factorization (NNMF). Among the abovementioned methods, NNMF is the most commonly used, due to its low computational complexity and to its nonnegativity constraints typical of muscle activation; evidence in literature has been provided supporting the choice of NNMF as the most reliable method for synergy analysis [15]. NNMF works by factorizing an original nonnegative data matrix **D** into nonnegative matrices **W** and **C** using simple multiplicative update rules that aim at minimizing iteratively the norm of the difference matrix between the original data matrix and its approximate reconstruction [17]. Despite different solutions having been proposed in recent literature to improve the performance of NNMF for synergy extraction [18, 19], the abovementioned implementation still constitutes the most widely used.

One of the main limitations of NNMF is that it assumes that the data are statistically independent [17, 20]; this makes the algorithm less effective in the factorization of data characterized by temporal correlation, such as the muscle activity detectable in pathological subjects [9, 11, 21] or during fast goal-directed movements [22]. From a practical point of view, a high correlation in the commands corresponds to a partial or complete temporal superposition/dependence, leading to a partly simultaneous recruitment of different muscle synergies; as a consequence, NNMF is not expected to have good performance in the accurate identification of the underlying synergy vectors, if they have highly correlated activation profiles [23].

Another challenging situation for the accurate identification of synergies through traditional factorization algorithms is the extraction of synergies that share the contribution of several muscles, such as in the activity of biarticular muscles that can contribute to more biomechanical subtasks; both shared muscles and a certain degree of correlation in the **C** coefficients are common features to be found in muscle synergy analyses, in particular when dealing with pathological subjects, and so, it is important to have more control on how the performance of the algorithm is affected by these characteristics of the experimental data.

The traditional NNMF employs random nonnegative initialization for **W** and **C** from uniform amplitude distribution; however, it has been shown that its performance can be improved when other initializations are taken into account [24–27]. It can be hypothesized that different initialization matrices might lead to the convergence to different local minima of the reconstruction error; in this context, it is important to evaluate whether a different initialization, based on the characteristics of the original data, leads to a local minimum corresponding to a more accurate representation of **W**, also in the presence of correlation among **C**. In addition, some studies have shown that the implementation of some sparseness constraints in the update rules can improve the performance of NNMF [28–30], suggesting the idea that inserting some sparseness constraints in the initialization data can affect and potentially improve the results of the factorization. Other studies have shown how initialization based on singular value decomposition (SVD) with nonnegativity constraints is able to improve the NNMF convergence properties guaranteeing a rapid reduction of the approximation error [24].

Based on this background, the aim of this study is to overcome the limitations of the commonly used random initialization in NNMF. In this regard, two alternative choices (SVD-based and sparse) are taken into account for comparison with the traditional implementation of NNMF. In our work, the method is applied in the framework of modular control of muscle coordination; in particular, we are interested in the correct identification of the spatial structure of the synergies, due to its task-representative role in our model. The different initializations are tested on a simulated dataset with controlled levels of correlation among activation coefficients and similarity in synergy composition, and their performance is compared to the traditional implementation of the NNMF algorithm; an additional validation has been carried out on artificially corrupted experimental data. We also investigated the local convergence error coming from different initializations, and we related it to the ability in accurately identifying **W**.

## 2. Materials and Methods

**Figure 1:**
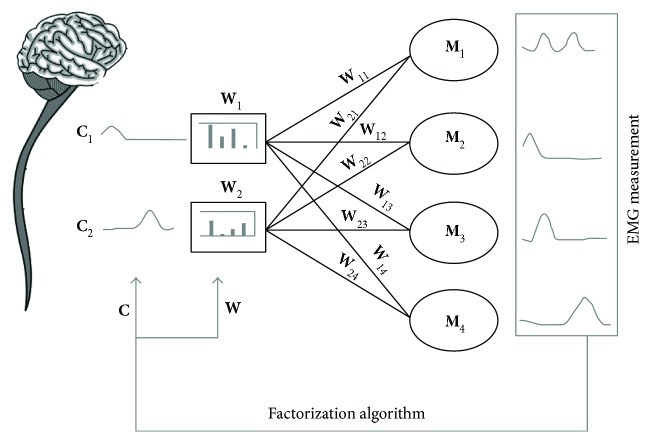
Synchronous muscle synergy model. In this graphical example, two different commands **C**, indicating the temporal activation coefficients, are sent to the mixing matrix **W**, consisting of four synergy vectors. The single command **C**_*i*_ is distributed to the muscle *M*_*j*_ according to the muscle weighting **W**_*ij*_. The inverse problem to be solved by a factorization algorithm is the accurate identification of the underlying structures **W** and **C** starting from the measurement of *D*.

Starting from the model of synchronous muscle synergies described in Figure 1 and (1), this section describes how the simulated sEMG dataset has been generated from synthetic muscle synergies **W** with controlled activation coefficients **C**. Each of these components has been simulated to reproduce different initial conditions for the generation of coordinated muscle activity.

In particular, synergy activation coefficients **C** have been simulated with different levels of temporal correlation, in order to reproduce a partial temporal superposition among activation coefficients. At the same time, synergy vectors **W** have been structured to reproduce the condition in which the contribution of a muscle to the whole coordination comes from more than one synergy. This technically implies a higher similarity between the corresponding original muscle synergy vectors. The contribution of these potentially challenging initial conditions is taken into account both separately and in combination, in order to assess their effect on the performance of the studied matrix factorization algorithms in the identification of the original modular structures.

After testing the behavior of the algorithm on a set of synthetic signals, the same analysis is carried out on a set of real multimuscle sEMG signals recorded from eight lower limb muscles during a pedaling task. The same signals are then artificially corrupted in order to induce a controlled temporal correlation among the synergy activation coefficients.

### 2.1. Generation of Simulated Data

Sets of simulated data were generated according to the following mathematical model of synchronous muscle synergies [15]:

(1) $$\begin{array}{c} D\left(t\right)=\overset{k}{\underset{n=1}{\sum }}C_{n}\left(t\right)W_{n}+\varepsilon \left(t\right),\;C\geq 0,W\geq 0, \end{array}$$

where **C**_(*k*, 5000)_ is the time-varying synergy activation coefficient matrix containing the activation of a single synergy in each row, *k* denotes the number of synergies underlying the data, **W**_(6, *k*)_ is the spatially fixed synergy matrix where each column is a synergy vector and contains six dimensions (i.e., the chosen number of muscles), and **D**_(6, 5000)_ is the simulated sEMG matrix that is a linear combination of **C** and **W**.

Nonnegative simulated data were generated with two basis vectors (i.e., synergies), six as data dimensions (i.e., muscles) each of which consists of 5000 data points (i.e., time samples). For every trial, activation coefficients were generated randomly with controlled correlation between them: in order to obtain such a controlled correlation between the generated data, multivariate normal random numbers were generated, with a zero mean and by using a symmetric square covariance matrix. For this purpose, the *mvnrnd* MATLAB function was used to generate correlated random variables that follow multivariate normal distribution. The mixed covariance terms were varied between 0 and 0.95 with steps of 0.05; this range has been set in order to span a wide range of correlation values, going from complete independence up to strong temporal dependence between activation coefficients, avoiding a complete correlation that would make it mathematically impossible to identify the underlying synergy vectors [15]. Data have then been scaled to obtain zero minimum, in order to ensure their nonnegativity and preserve the controlled level of correlation.

**Figure 2:**
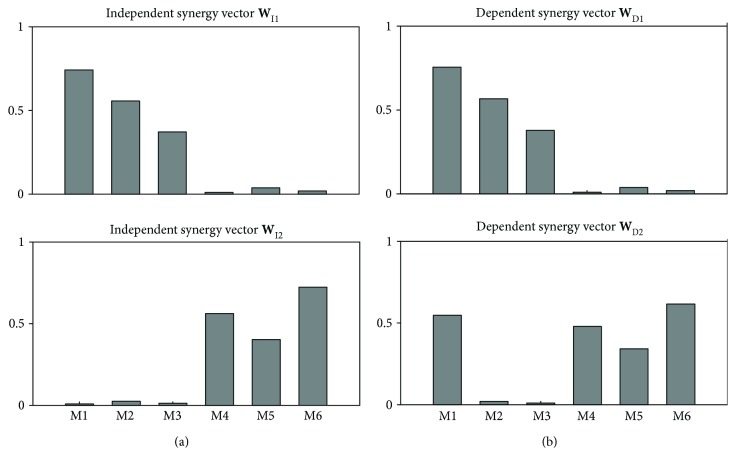
Simulated muscle synergy vectors: (a) the independent synergy vectors **W**_I_, in which each muscle significantly contributes to only one synergy; (b) the dependent synergy vectors **W**_D_, in which the contribution of muscle M1 is comparable between the two synergies.

The simulated datasets were generated with respect to the following two cases:

**Case 1**

(independent synergies (**W**) and dependent activation coefficients (**C**)). In this dataset, data were simulated using independent synergies (Figure 2(a)) and activation coefficients with controlled correlation values between 0 and 0.95.

**Case 2**

(dependent synergies (**W**) and dependent activation coefficients (**C**)). This dataset is generated by using the same properties as in Case 1 but using dependent synergies, in which a single muscle is shared between synergies as shown in Figure 2(b).

### 2.2. Real Data

Real EMG data were used for an additional qualitative test. sEMG data used in this study are taken from a single subject among those enrolled in [31]; details regarding the protocol and sEMG processing can be found there. Briefly, data were recorded from eight lower limb muscles (*gluteus maximus*, *biceps femoris*, *gastrocnemius medialis*, *soleus*, *rectus femoris*, *vastus medialis* and *lateralis*, and *tibialis anterior*) during a 2 min long unconstrained pedaling task at 60 rpm on a cycle ergometer. EMG data were bandpass filtered (20 Hz–400 Hz), full-wave rectified, and low-pass filtered (5 Hz) to obtain the linear envelope. These experimental EMG envelopes underwent a classical muscle synergy extraction via NNMF to obtain the underlying **W**_EXP_ and **C**_EXP_. In order to analyse the performance of the initialization methods described in this paper, the extracted independent muscle activation shown in [31] is artificially corrupted by making them temporally dependent; the four activation profiles were time shifted to obtain the maximum degree of correlation, obtaining the correlated activation coefficients **C**_CORR_. The modified EMG envelope matrix **D**_CORR_ was thus constructed as **W**_EXP_ **C**_CORR_. Then all the initialization methods described in the following section were applied on **D**_CORR_, aiming to identify **W**_EXP_.

### 2.3. Nonnegative Matrix Factorization

NNMF factorizes the given nonnegative data **D**_(*M*, *N*)_ into two matrices **W** and **C** such that **D** ≈ **W** **C**, where **W**_(*M*, *k*)_ is the synergy matrix, **C**_(*k*, *N*)_ is the activation coefficient matrix, *M* is the number of muscles, *N* is the number of time samples, and *k* is the number of extracted synergies. We used the original version of the algorithm implementing the multiplicative update rules introduced in [17], defined by the following:

(2) $$\begin{array}{c} W_{ik}\leftarrow W_{ik}\frac{{\left({DC}^{T}\right)}_{ik}}{{\left({WCC}^{T}\right)}_{ik}},\\ C_{kj}\leftarrow c_{kj}\frac{{\left(W^{T}D\right)}_{kj}}{{\left({WW}^{T}C\right)}_{kj}}. \end{array}$$

The aforementioned update rules are applied until the difference in the Frobenius norm ‖**D** − **W** **C**‖_Fr_ between two successive iterations is lower than a certain threshold.

### 2.4. Initialization Techniques

From now on, only the different initialization techniques of the **W** matrix will be described, while the **C** matrix of the activation coefficients is always initialized with values taken from a uniform amplitude distribution between 0 and 1 (MATLAB *rand*).

The most used method for the initialization of the **W** matrix in NNMF considers the elements of the initialization matrix to be realizations of a random uniform amplitude distribution in the range [0, 1]. Data have been generated by using MATLAB *rand*, and this classical initialization will be referred to as RAND from now on.

Another initialization method is based on singular value decomposition [24]. SVD has been applied to the data matrix **D**_(*M*, *N*)_ in order to obtain the following representation:

(3) $$\begin{array}{c} D=WSC\;with\;W,S,C\geq 0, \end{array}$$

where **S**_(*k*, *k*)_ is the matrix of the singular values. We achieved nonnegativity in the initialization of **W**_(*M*, *k*)_ and **C**_(*k*, *N*)_ by initializing them according to the procedure described in [24], which provides a low-rank nonnegative SVD initialization of **W** and **C**. This initialization will be referred to as NSVD.

The third initialization technique imposes a sparse structure in the spatial organization of the synergy vectors. The **W** matrix was first initialized with values taken from a uniform random amplitude distribution in the range [0, 0.05]. Subsequently, one random element in each synergy has been set to a random uniform value in the range [0.7, 0.8]; these values have been chosen arbitrarily in order to simulate the activation of just one muscle for each synergy and to obtain an extremely sparse synergy initialization (i.e., only one active muscle in each synergy vector). We will refer to this initialization as SPARSE.

### 2.5. Assessment of the Performance in Synergy Identification

In order to assess the performance of the three analysed initialization techniques on the simulated datasets, a quality of reconstruction (QR) parameter is used. QR calculates an average similarity based on the cosine of the angle between the generated muscle synergies and the synergies extracted by each algorithm, as often done in previous studies [32]. The QR gives a value between 0 and 1, with 1 indicating the highest similarity between extracted muscle synergies and the simulated ones (i.e., parallel vectors in the M-dimensional space).

For simulated data, QR has been computed for all the initialization techniques across a wide range of correlation levels (using 20 steps from 0 to 0.95). The average QR value over 100 runs has been computed between the original muscle synergies, synthetically generated, and the synergies extracted by NNMF.

We used a Kruskal-Wallis analysis to check the effect of initialization technique and correlation level on the QR parameter.

## 3. Results

### 3.1. Simulated Data

**Figure 3:**
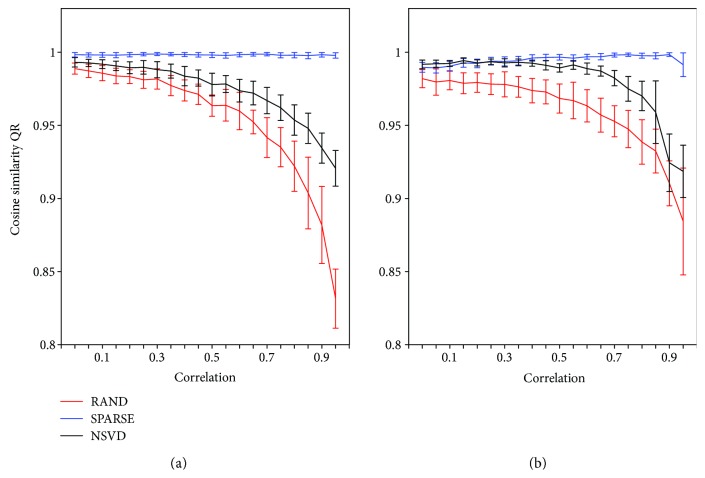
Performance of NNMF with three different initialization techniques. Average cosine similarity between the synergies extracted from an initialization technique and the original simulated synergies **W**. For each of the 20 correlation levels between the activation coefficients **C**, the performance is reported as median ± median absolute deviation across 100 realizations. (a) Data generated with independent synergy vectors **W**_I_. (b) Data generated with dependent synergy vectors **W**_D_.

**Figure 4:**
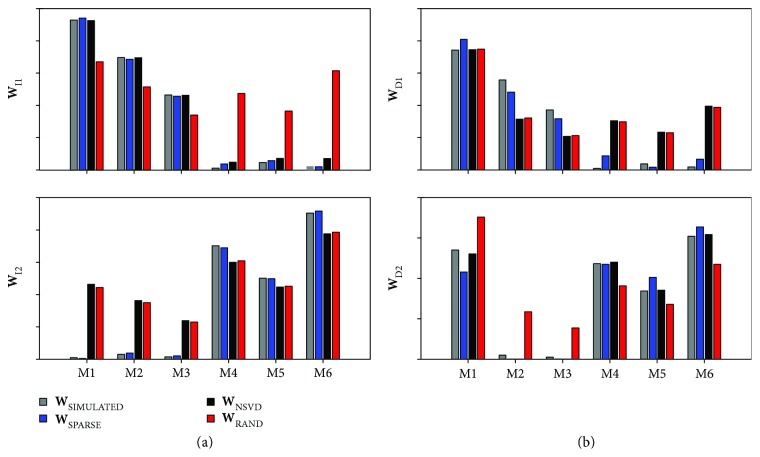
Example of synergy extraction with different initialization techniques with a correlation level of 0.9: (a) independent synergy vectors **W**_I_; (b) dependent synergy vectors **W**_D_. Only SPARSE is able to accurately estimate **W** in both cases.

Figure 3 shows the results regarding synergy identification obtained by each method for data generated both with independent and dependent synergies and correlated activation coefficients. Kruskal-Wallis test shows a general strong effect of correlation in the correct identification of **W** (*p* < *E* − 10). Moreover, QR is differently affected by the initialization technique, with post hoc analysis indicating that QR_SPARSE_ > QR_SVD_ > QR_RAND_ (*p* < *E* − 5).  Figure 4 shows an example of synergy identification with all initialization techniques when data are generated with dependent and independent synergies with a level of correlation of 0.9; it can be seen how the performance of RAND decreases dramatically when correlation among muscle activation coefficients is high, while NSVD keeps an acceptable performance, although SPARSE maintains better overall performance.

**Figure 5:**
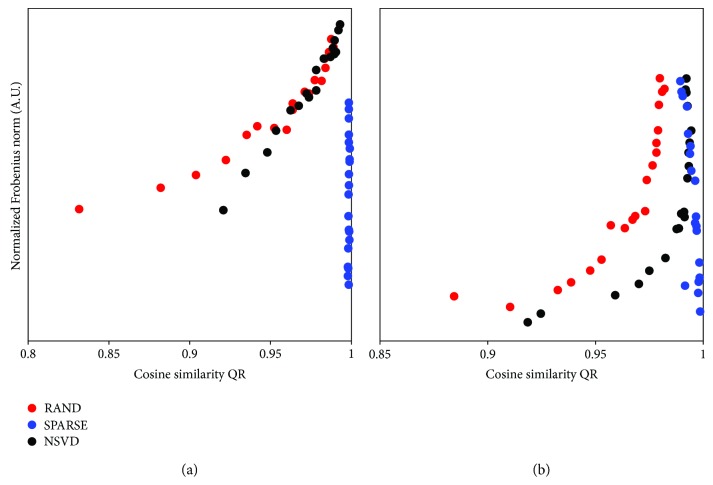
Relationship between the estimation quality QR and the approximation error of NNMF (i.e., Frobenius norm) with different initialization techniques. (a) Independent synergies **W**_I_. (b) Dependent synergies **W**_D_. In both cases, for RAND and NSVD initialization, higher synergy estimation accuracy QR is obtained with a higher approximation error via NNMF, while SPARSE initialization is not dependent on the NNMF convergence error.

In order to check whether a more accurate identification of the underlying synergy vectors **W** (i.e., higher QR) is related to a lower convergence error of NNMF (i.e., the Frobenius norm (FN)), we qualitatively analysed the relation between these quantities across different levels of correlation for each initialization technique. The QR-FN curve was built and shown in Figure 5 for both the dependent and independent synergies, considering the median QR and FN values across the 100 realizations for all the correlation levels and all three initializations; as shown in Figure 5, a 20-point trace was obtained for each initialization technique.

### 3.2. Real Data

**Figure 6:**
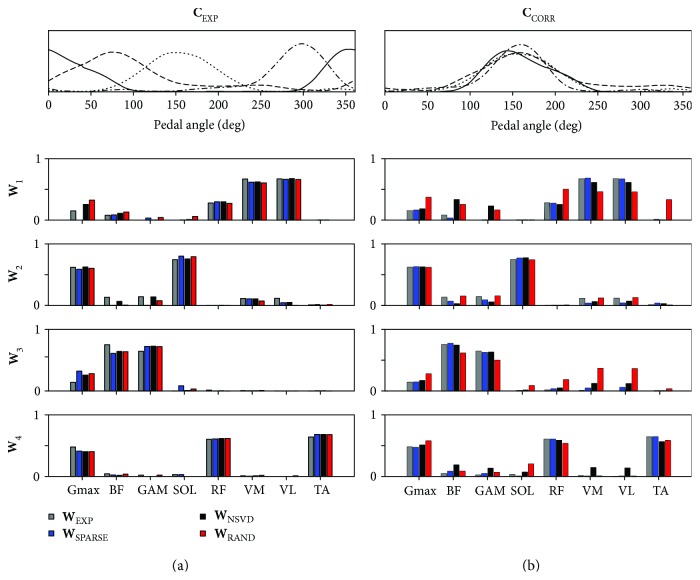
Decomposition of experimental EMG data recorded during pedaling. (a) Decomposition of matrix **D** = **W**_EXP_∗**C**_EXP_; synergy activation coefficients **C**_EXP_ (upper) and synergy vectors obtained with the three different initialization techniques, together with the original **W**_EXP_. (b) Decomposition of matrix **D** = **W**_EXP_∗**C**_CORR_; synergy activation coefficients **C**_CORR_ (upper) and synergy vectors obtained with the three different initialization techniques, together with the original **W**_EXP_.

Four synergies were extracted as in [31].  Figure 6 (left column) shows that the muscle synergies extracted by each method are very similar both in terms of **W** and **C** when the original data are decomposed by the three initialization techniques. This result is in accordance with those obtained on the simulated dataset, as in the data both partially dependent synergy vectors (contribution of RF muscle is shared among 2 identified synergies) and independent muscle activation coefficients (as identified by the clearly separated peaks of activity of the four different synergies) contribute to the measured muscle coordination. The high correlation was achieved by delaying the single activation coefficients with respect to the lag corresponding to the maximum of the cross-correlation function. The correlation levels obtained with this procedure are reported in Table 1. In Figure 6(b), the decrease in performance of RAND and NSVD is clearly visible and confirmed by the QR values indicated in Table 2.

## 4. Discussions

The main aim of this study was to assess the effect of three different initialization strategies for NNMF. Ad hoc synergies, both orthogonal or with spatial dependencies, were generated, in order to evaluate the quality of their estimation with respect to different initialization choices. Temporal activation coefficients were simulated with different levels of correlation, to test NNMF behavior under challenging conditions. Our comparative study suggests that NNMF performances are not independent from the initialization and that the traditional random initialization yields worse performances than other methods, with the most significant differences in the case of datasets with a high degree of correlation.

In our simulation study, we found that NNMF accurately identifies muscle synergies when the correlation between activation coefficients was low, regardless of the initialization choice. However, high correlation between the activation coefficients caused a dramatic worsening of the synergy identification accuracy, if the first choice for **W** is not well structured. RAND initialization has the worst performance in the presence of dependency among synergy vectors (i.e., one muscle is shared between two synergies) or of correlation among activation coefficients; considering this, this kind of initialization structure does not represent the optimal choice when the degree of correlation or the spatial structure is not known. On the other hand, NSVD and SPARSE have been shown to yield good quality in the identification of the simulated synergy vectors for all the correlation values tested in this work and for all the different sets of motor modules. These two methods have shown similar performance, with some advantages of SPARSE for extremely high correlation values; however, the NSVD initialization requires some calculations on data before the NNMF algorithm, introducing an additional step in the extraction procedure that can affect the computational complexity as well as the integrity of the data structure, given the fact that the **S** matrix of the singular values is not considered in the algorithm. Considering these results, we hypothesize that SPARSE initialization might be the best choice for seeding the NNMF algorithm, due to its performances and its negligible computational cost. By adopting this initialization procedure, we infer that the results of modular motor control analyses can be improved significantly, in particular in experiments dealing with challenging muscle activation patterns characterized by a high level of muscle cocontraction [33].

In this work, we have simulated a modular motor control scheme characterized by the activity of two synergies; in literature, however, it has been shown that most common tasks are well described by the activation of 3 to 5 synergies [4–9]. Even though a specific study on experimental data is needed for a full validation, preliminary results from our analysis on artificially corrupted experimental data suggest that our interpretations are still valid when the rank of the model is higher than two, and data are characterized by mixed levels of correlation among activation coefficients and similarity among synergy vectors; however, a systematic experimental validation of our results is critical for application in the experimental framework, when no a priori knowledge on the number of synergies or on the degree of spatial or temporal correlation can be assumed.

Our results refer to the correct identification of the **W** matrix, because we hypothesized that after the correct identification of one component of the spatiotemporal coordination of muscles, it is straightforward to extract the other by inverting the matrix equation or using some reconstruction algorithm; the same observation is valid for the problem of identifying the correct **C** matrix, as some motor control theories hypothesize a modular structure with invariant **C** as primitives [34]. In the framework of muscle synergy analysis, it can be logical to aim for a maximization of the performance in the estimation of one of the two components, in contrast with the minimization of the approximation error for the original data matrix **D**. As qualitatively shown in our results, the minimum approximation error for **D** is not always related to the maximum quality in the identification of **W**; on the contrary, a counterintuitive conclusion has been drawn from this preliminary analysis, where a higher approximation error corresponds to a more accurate identification of the underlying synergies **W**. This means that there is the need for a more detailed investigation on the clinical meaning that it is attributed to the different component of the muscle synergy model, in order to develop experimental and extraction methods aiming for the most correct extraction of meaningful information.

Recently, features of the modular motor control schemes have assumed more clinical importance. Our results show that the typical implementation of NNMF used in literature does not show consistent results across the whole range of possible features of sEMG data in clinical experiments; given this, it is clear how it is important to have deeper knowledge on NNMF behavior, when dealing with typical physiological data. Without clarifying upon the previous point, it is extremely difficult to assign an objective and quantitative meaning to the interpretation of the structure of the synergy vectors.

In this work, all the analyses have been carried out without the need to select the correct number of synergies; the problem of a correct choice of the rank of NNMF approximation is still an open issue that must be solved for improving the robustness of this kind of analysis [19, 21]. Although recent studies tried to solve this ambiguity [35], no standard methods have been proposed before, and it is important to understand how the different initialization strategies affect the identification of the correct number of motor modules, to allow for a complete knowledge about the CNS control strategies.

## 5. Conclusions

This study assessed the performances of different initialization techniques for NNMF, when different levels of correlation characterize the temporal activation coefficients. The results demonstrate that the performance of NNMF with the classical random initialization decreases dramatically when correlation among synergy activation coefficients increases, both for independent and dependent synergy vectors. When the aim is the accurate identification of the spatial composition of motor control, a sparse initialization to the synergy vectors **W** significantly outperforms the other initialization techniques in the presence of correlation among synergy activation coefficients. A proper shaping of the initial matrix can thus significantly improve the convergence properties of the NNMF algorithm for the accurate extraction of muscle synergies, regardless of the statistical distribution of the matrix of the synergy activation coefficients. These findings suggest that a sparse initialization is preferable, particularly in those experimental conditions where biomechanical or neural constraints impose a strong muscle cocontraction. The higher estimation accuracy might help improving the neuromechanical and clinical significance of muscle synergy analysis.

## Figures and Tables

**Table 1:** Correlation between muscle activation coefficients before (**C**_EXP_) and after the artificial intervention (**C**_CORR_) to make them temporally correlated.

**C**_EXP_/**C**_CORR_	**C**1	**C**2	**C**3	**C**4
**C**1	—	0.13/0.86	−0.42/0.91	−0.12/0.87
**C**2	—	—	−0.1/0.87	−0.48/0.89
**C**3	—	—	—	−0.38/0.88
**C**4	—	—	—	—

**Table 2:** Cosine similarity QR between pairs of homologous synergy vectors extracted via different initialization methods. Similarity values are presented when the extraction is performed on the original and on the artificially corrupted EMGs, built as **D** = **W**∗[**C**_EXP_ or **C**_CORR_].

**C**_EXP_/**C**_CORR_	SPARSE	NSVD	RAND
**W**1	0.99/0.99	0.99/0.94	0.98/*0.87*
**W**2	0.97/0.99	0.99/0.99	0.98/0.98
**W**3	0.98/0.99	0.98/0.99	0.97/*0.70*
**W**4	0.99/0.99	0.99/0.97	0.99/0.97
